# Supplementary material for: Disentangling the Functional Role of Fungi in Cold Seep Sediment
Source: Microbiol Spectr. 2023 Mar 13;11(2):e01978-22. doi: 10.1128/spectrum.01978-22 (PMC10100914; doi:10.1128/spectrum.01978-22)
Supplement: Supplemental file 1 — Supplemental material. Download spectrum.01978-22-s0001.pdf, PDF file, 1.4 MB [file spectrum.01978-22-s0001.pdf]

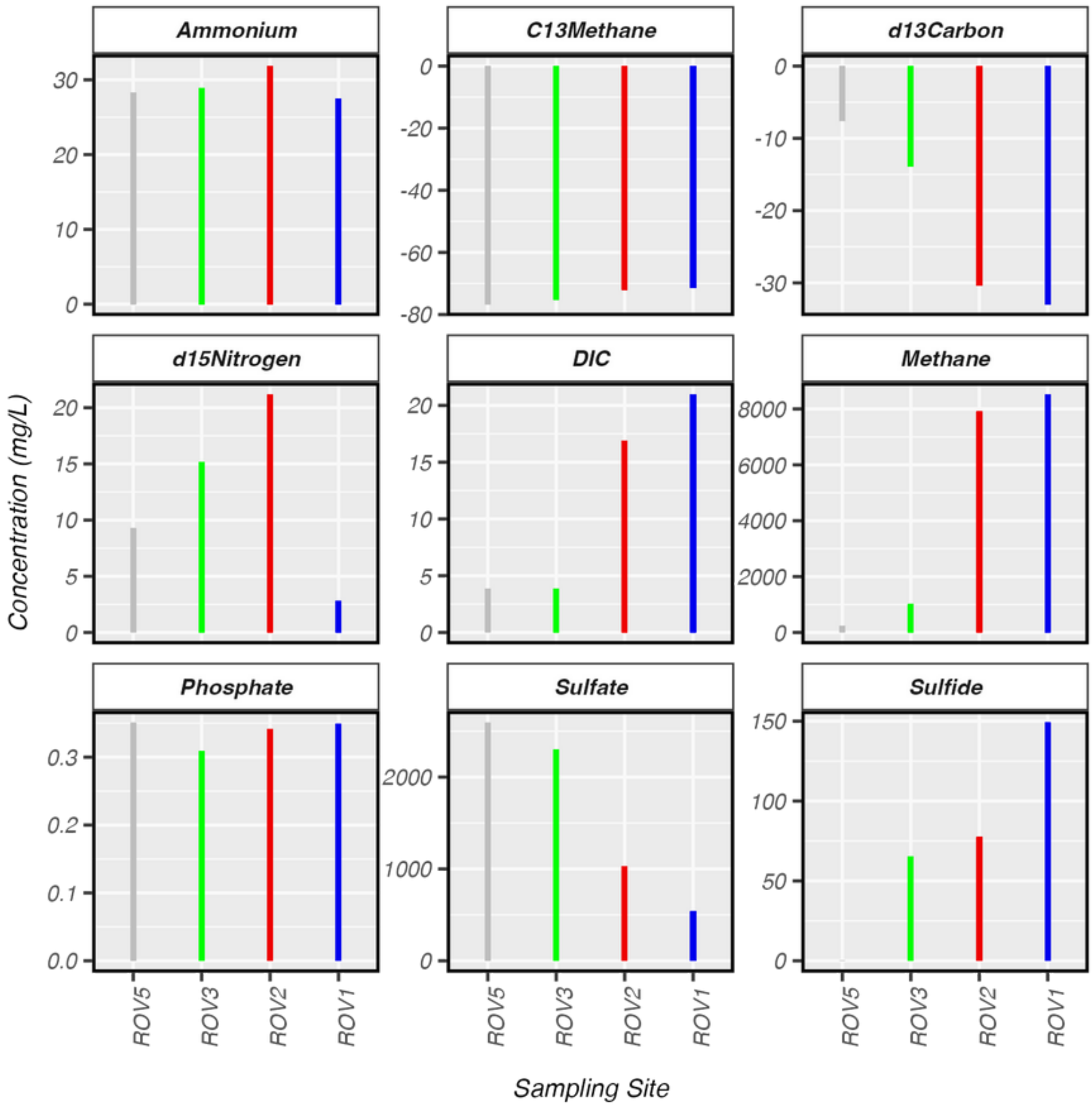

Figure S1 | Average concentration in units mg/L of all abiotic factors measured across all sites.

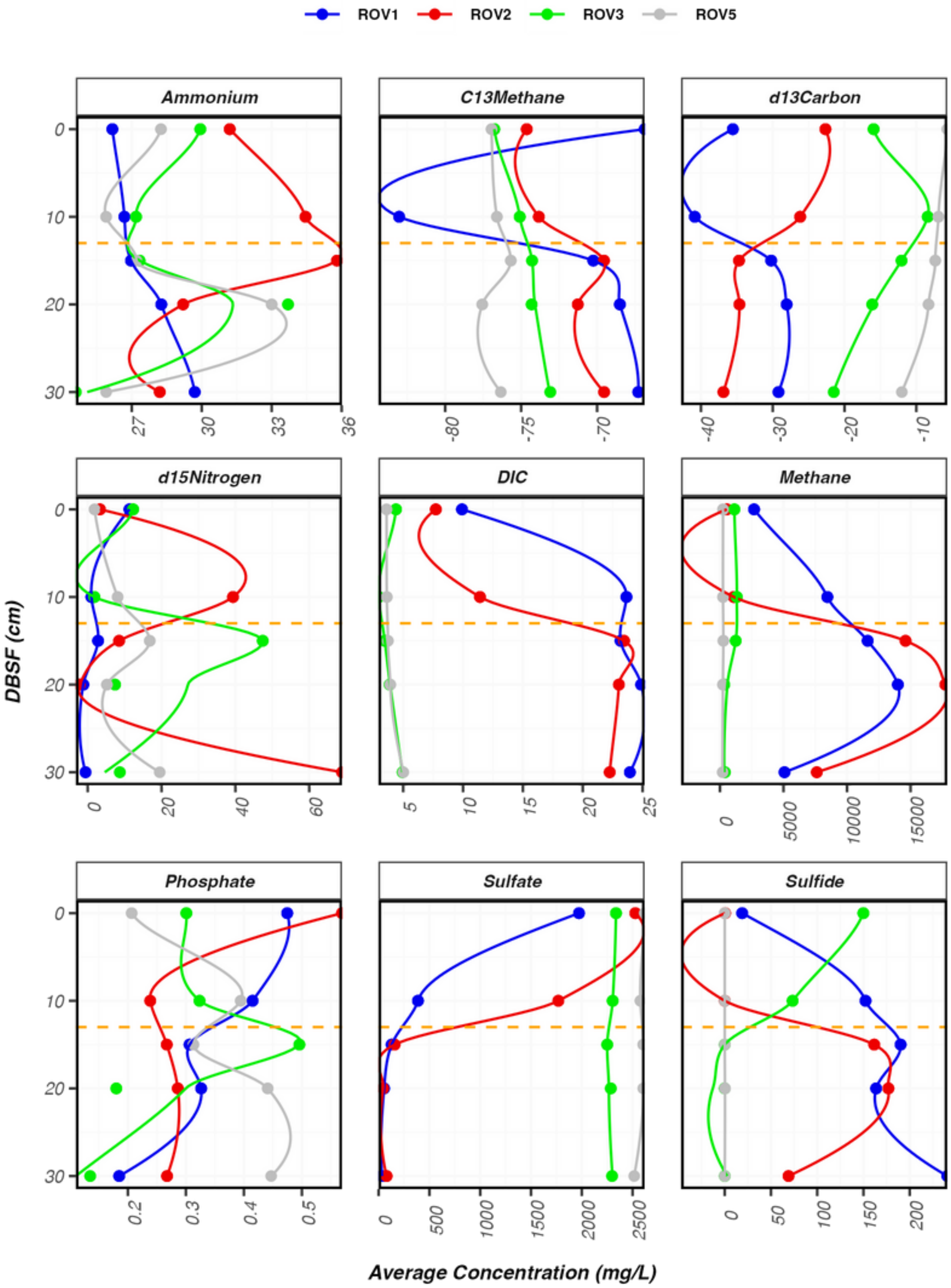

Figure S2 | Vertical depth profile (Depth Bellow Sea Floor, DBSF, measured in centimeters, cm) of the concentration of all abiotic factors (milligram per liter mg/L) measured in all sites of this study.

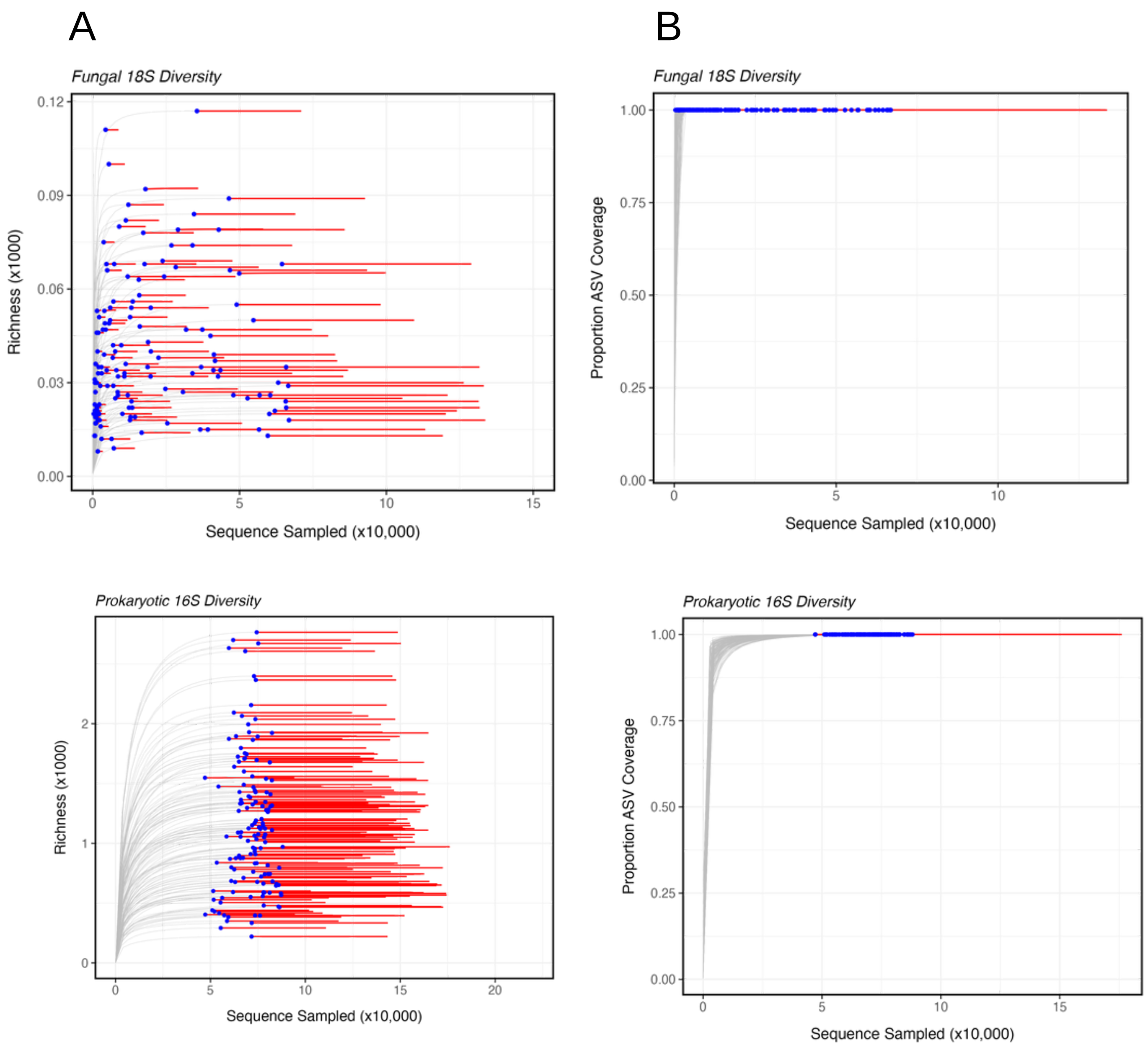

Figure S3 | A) Sample-based rarefaction curves of both 16S and fungal 18S sequences across 10,000 iterations as inferred with the "iNEXT" R package B) Coverage rarefaction curves showing 100% (1.0) coverage achieved in all samples for both 16S and fungal 18S.

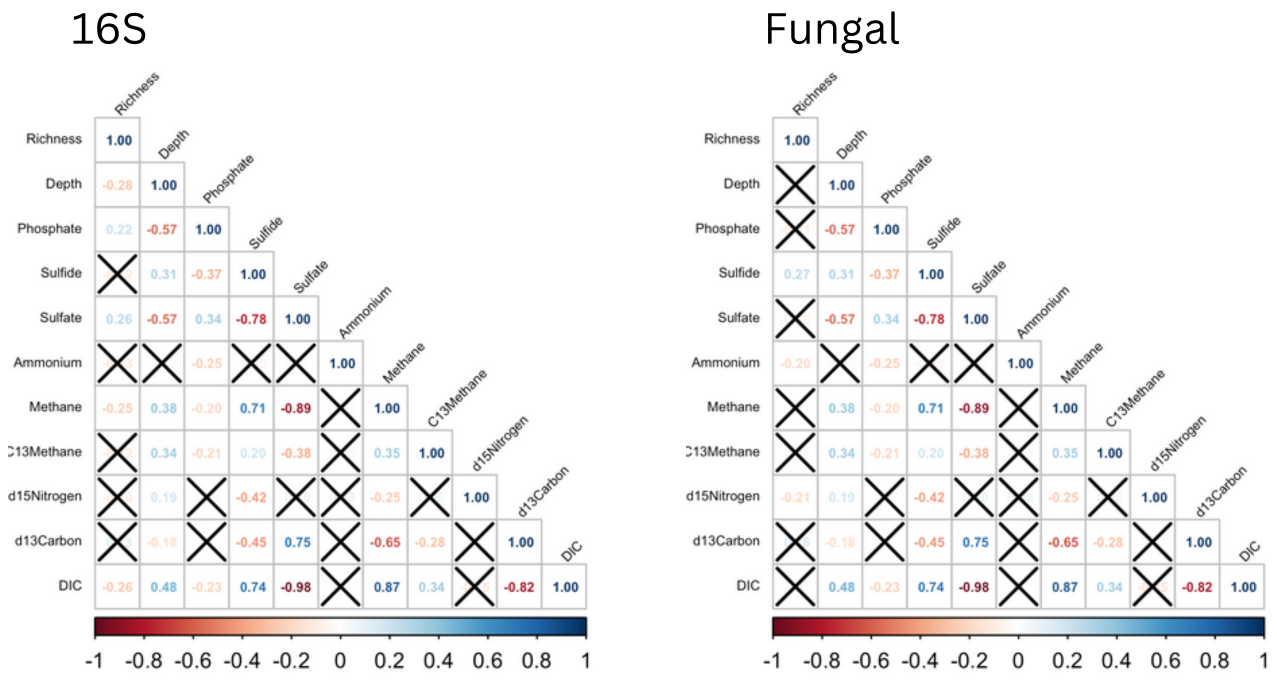

Figure S4 | Pearson correlation association matrix of different abiotic factors and richness. Non-significant values are canceled with an X sign.

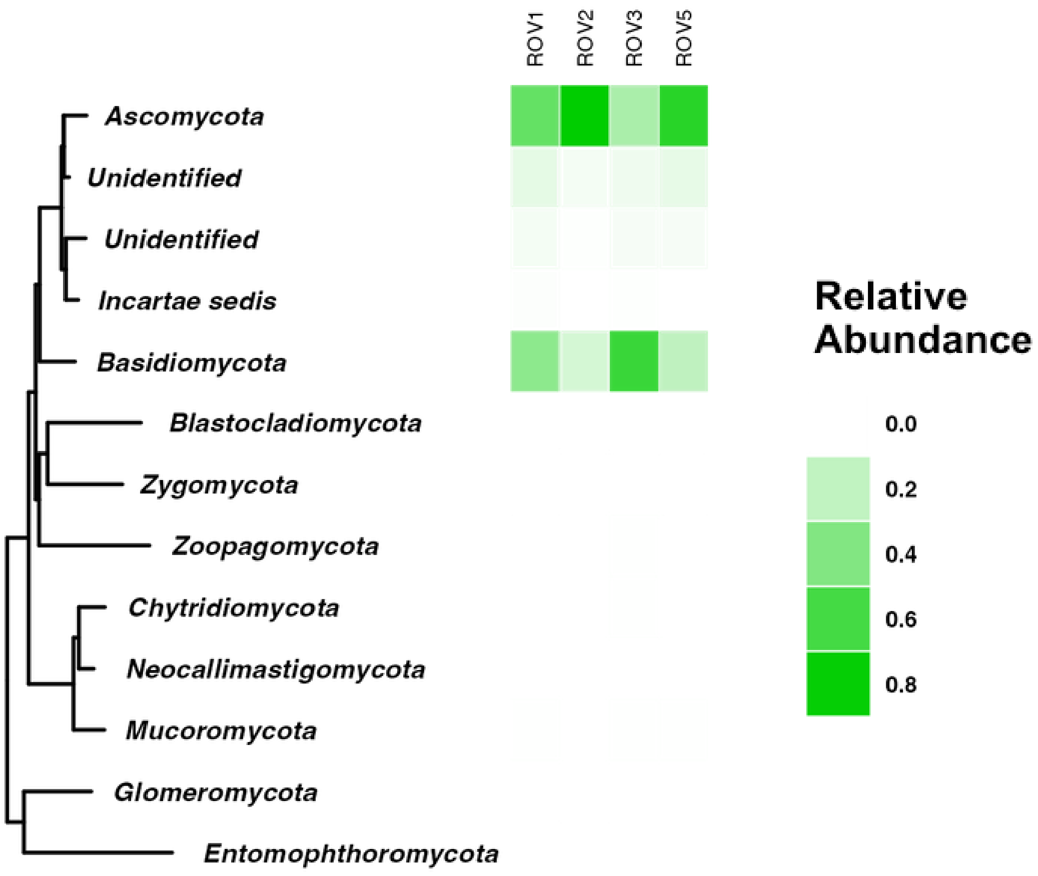

Figure S5 | Community composition analysis using 18S fungal and 16S prokaryotic primers. Relative-abundance phylum-level heatmap of different sampling sites (Generated by 18S MAFFT alignment and plotted using ggtree packaged in R)

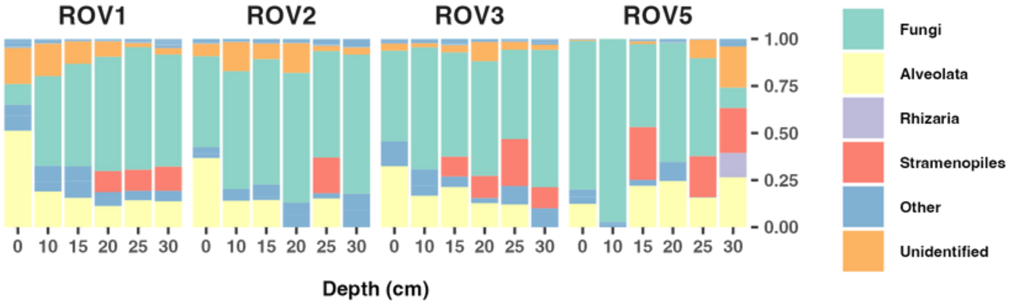

Figure S6 | Average relative abundance bar-chart of fungal 18S rRNA gene region as compared to other microeukaryotes in different depths and sites (Depth Bellow Sea Floor in centimeters).

A

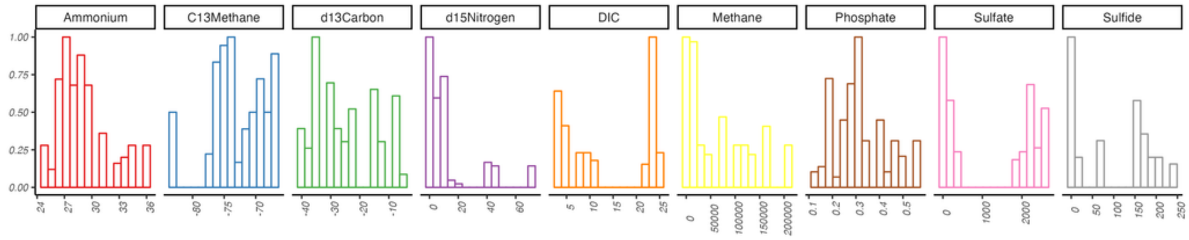

B

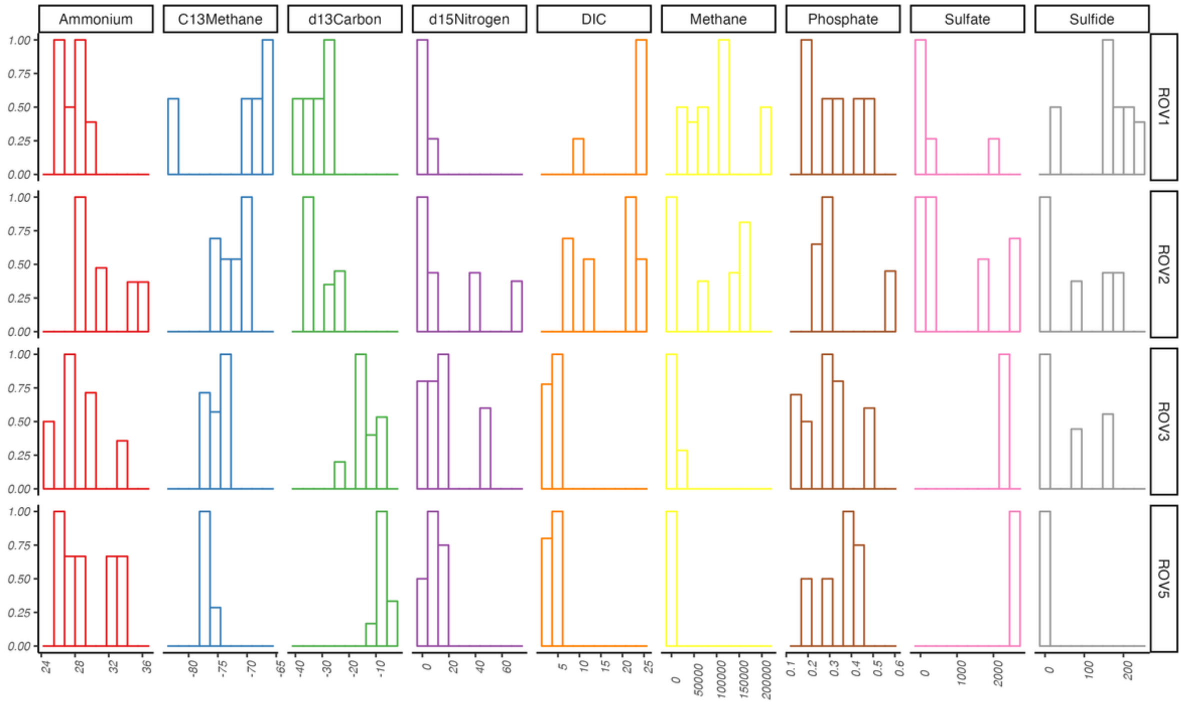

Figure S7 | Distribution histogram of abiotic factors A) Across all samples B) Sample-specific

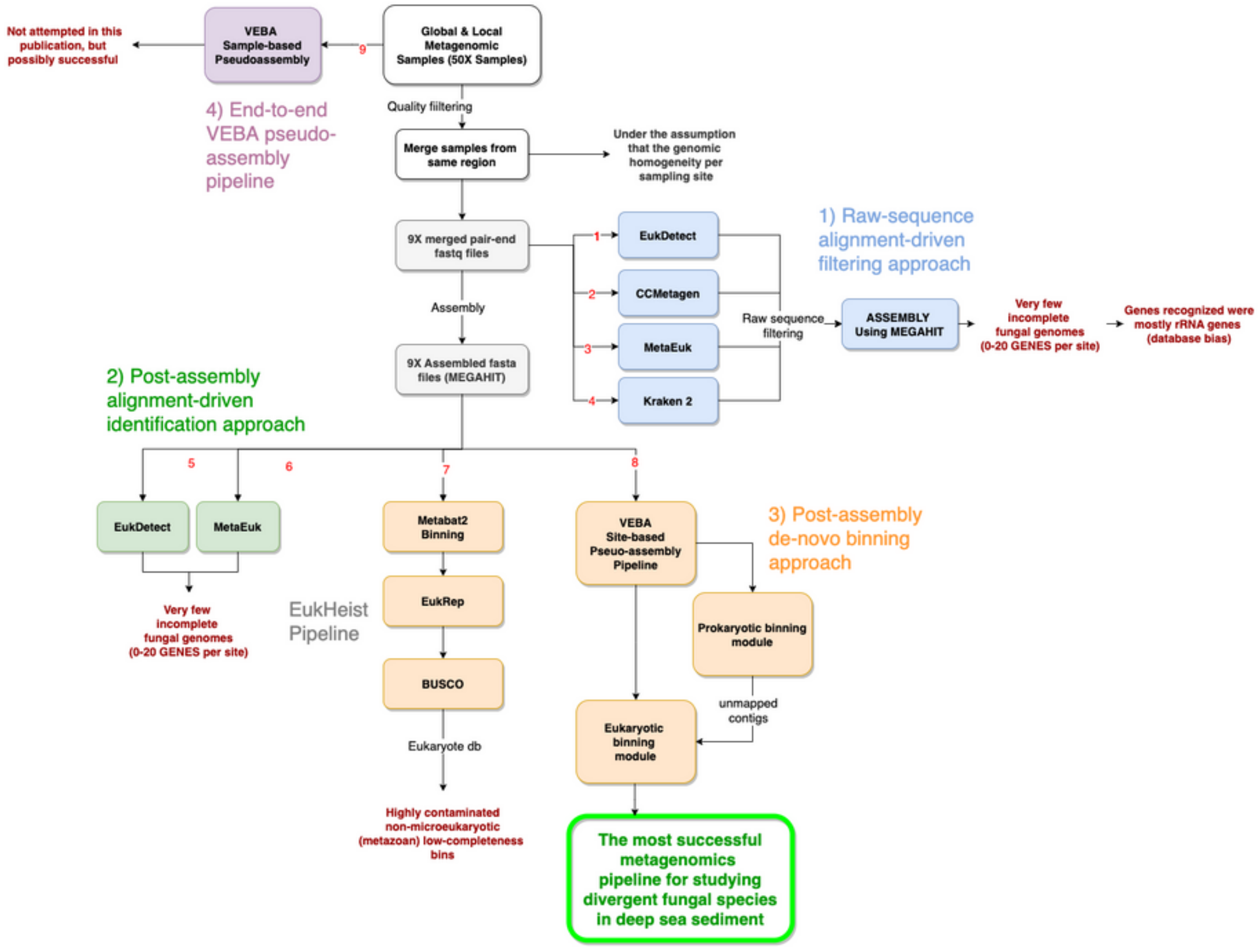

Figure S8 | Failed strategies for scavenging fungal hydrophobin, cytochrome p450, and ligninolytic enzymes in a global metagenomic dataset. With the most successful being the VEBA strategy.

Sample Site	Data Type	Status	Number of Samples	DOI	Data Source	Accession Number
JL	Metatranscritptome	Raw reads	4	10.1038/s41467-022-32503-w	SRA	PRJNA831433
HAI	Metatranscritptome	Raw reads	1	10.1038/s41467-022-32503-w	SRA	PRJNA831433
GM	Metatranscritptome	Assembly	3	10.1128/mSystems.00091-19	IMG JGI	3300008633
EGM	Metagenome	Raw reads	3	10.1038/s41467-019-09747-2	SRA	PRJNA485648
ENP	Metagenome	Raw reads	14	10.1111/1462-2920.15658	SRA	PRJNA390944
HM	Metagenome	Raw reads	2	10.1594/PANGAEA.861267	SRA	PRJNA248084
MSA	Metagenome	Raw reads	1	10.3389/fmicb.2018.02917	SRA	PRJNA431796
SMM1	Metagenome	Raw reads	1	10.3389/fmicb.2018.02918	SRA	PRJNA431797
SMM2	Metagenome	Raw reads	2	10.1128/mBio.00530-17	SRA	PRJNA326769
SB	Metagenome	Raw reads	7	10.1038/s41467-020-19648-2	SRA	PRJNA598277
WGM	Metagenome	Raw reads	7	10.1128/mBio.01814-23	ENA	PRJEB32776
HAI	Metagenome	Raw reads	21	This study	SRA	PRJNA849592

Table S1 | Summary of global metagenomic and metatranscriptomic datasets used in this study.
